# A Systematic Evaluation of the SARS-CoV-2 Vaccine-Induced Anti-S-RBD-Ig Response in a Population of Health Care Workers

**DOI:** 10.3390/vaccines11091467

**Published:** 2023-09-07

**Authors:** Viktoria Hentschel, Cornelia Horsch, Benjamin Mayer, Annsophie Thies, Will Qian, Joris Kroschel, Thomas Seufferlein, Lukas Perkhofer, Martin Müller

**Affiliations:** 1 Department of Internal Medicine I, Ulm University Hospital, 89081 Ulm, Germany; viktoria.hentschel@uniklinik-ulm.de (V.H.); annsophie.thies@uni-ulm.de (A.T.); will.qian@uni-ulm.de (W.Q.); thomas.seufferlein@uniklinik-ulm.de (T.S.); lukas.perkhofer@uniklinik-ulm.de (L.P.); 2 Institute for Epidemiology and Medical Biometry, Ulm University, 89075 Ulm, Germany; chorsch@rheinahrcampus.de (C.H.); benjamin.mayer@uni-ulm.de (B.M.); 3 Central Department of Clinical Chemistry, Ulm University Hospital, 89081 Ulm, Germany; joris.kroschel@uniklinik-ulm.de; 4 Institute of Molecular Oncology and Stem Cell Biology, Ulm University Hospital, 89081 Ulm, Germany

**Keywords:** SARS-CoV-2, humoral immune response, mRNA vaccines

## Abstract

In the wake of the COVID-19 pandemic, the novel class of mRNA vaccines has been granted first-time approval for active immunization against SARS-CoV-2 alongside the already established viral vector-based vaccines. In this prospective single-center study, we set out to determine the vaccine-induced humoral immune response in a population of 1512 health care employees after the second and third vaccination, respectively. Anti-SARS-CoV-2 receptor-binding domain (RBD) and nucleocapsid antigen antibody concentrations were assessed using commercially available immunoassays. We could show that, in particular, young study subjects aged below 30 years, as well as those with a prior SARS-CoV-2 infection, developed significantly higher antibody concentrations. Our data further suggest that being in physically close contact with formerly SARS-CoV-2-positive people positively affects the post-vaccination response. Surprisingly, study subjects with a BMI > 30 produced the highest anti-S-RBD Ig antibody levels if they had recently received their third vaccination. Also, heterologous dual vaccine regimens consisting of a BNT162b2 and ChAdOx1 n-CoV-19, a homologous triple combination of BNT162b2, and an application of mRNA-1273 as the third vaccine, were most efficient at eliciting a humoral immune response. Our study substantiates existing evidence, but beyond that, scrutinizes the impact of vaccine agents and their respective combinations, as well as different time intervals on humoral immunogenicity.

## 1. Introduction

SARS-CoV-2 is a novel human pathogenic coronavirus that can cause severe pneumonia and acute respiratory distress syndrome (Corona Virus Disease-2019, COVID-19) especially in elderly and immunocompromised people. Due to its high contagiosity, SARS-CoV-2 evoked a one-in-a-century pandemic which has claimed several millions of deaths to date. The most common vaccines administered in the Western world were the new class of mRNA vaccines BNT162b2 (Comirnaty—Pfizer, New York City, NY, USA/BioNTech, Mainz, Germany), mRNA-1273 (Spikevax—Moderna, Cambridge, MA, USA/NIAID, Rockville, MD, USA), and the adenoviral vector-based vaccine ChAdOx1 n-CoV-19 (Vaxzevria—University of Oxford, UK/AstraZeneca, Cambridge, UK) [1]. The internationally recommended active immunization schedules include at least two vaccinations to build up a basic protective immunity, followed by a “booster” vaccination. Mostly due to an initial shortage in vaccine doses, prime-boost immunization series were not always completed with the same type of vaccine (homologous regimen), thus often incorporating vaccines with different modes of action or supplied from different manufacturers (heterologous regimen) [2,3]. Increasing evidence suggests that combining different vaccines may be associated with greater immunogenicity [4,5], resulting in increased B and T cell reactivity and antibody production [6,7,8]. In particular, the administration of either of the mRNA vaccines, mRNA-1273 or BNT162b2, following initial immunization with ChAdOx1 n-CoV-19, has been shown to induce a stronger immune response compared with a homologous scheme of ChAdOx1 n-CoV-19 [9]. Furthermore, the humoral response to vaccination against SARS-CoV-2 has been shown to be modulated by smoking [10,11], obesity [12], age-related immune dysfunction [13], female sex [14], immunosuppressive medications [15], cross-reactivity with other seasonal coronaviruses [16], or influenza [17].

To date, a multitude of clinical studies on the post-vaccination antibody response is available, mostly focusing on the general safety issues of the new vaccines, the immunogenicity and reactogenicity in highly selected patient populations, and the long-term monitoring of antibody levels following specific vaccine regimens or natural infection [18,19]. Foremost, this study aims at exploring the humoral immunogenicity of the three most prevalent SARS-CoV-2 vaccines in Central Europe—BNT162b2, mRNA-1273, and ChAdOx1 n-CoV-19—with a focus on the most frequent combinations as well as the sequence of vaccines by probing anti-S-RBD Ig antibody concentrations in response to the second and third vaccination in a population of predominantly healthy individuals. Moreover, we take into account the basic demographic features and selected elements of the study subjects’ medical and social histories, since these variables can have profound repercussions on the output of the humoral immune system.

## 2. Materials and Methods

### 2.1. Study Population

**Figure 1:**
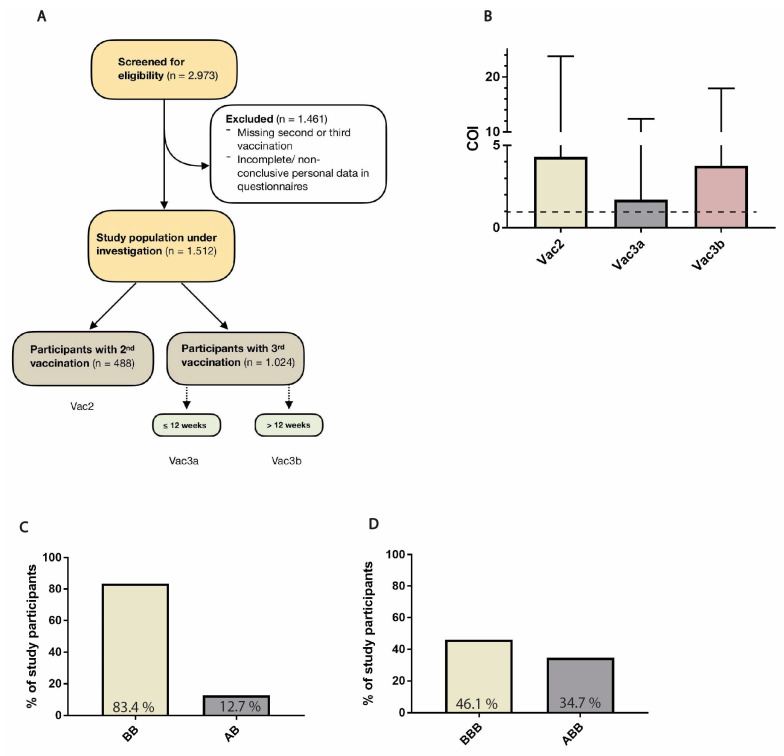
CONSORT diagram: The flow chart illustrates the process of the enrollment and subdivision of study subjects according to their vaccination status into groups Vac2, Vac3a, and Vac3b, respectively (**A**). A history of native SARS-CoV-2 infection is indicated by a COI ≥ 1. Mean COI values and standard deviations (represented by error bars) of Vac2, Vac3a, and Vac3b study subjects are plotted in (**B**). The two most prevalent vaccine combinations in double- and triple-vaccinated study subjects are displayed in (**C**,**D**), respectively. COI, cut-off index; B, BNT162b2/BioNTech; A, ChAdOx1 n-CoV-19/AstraZeneca.

The study population was recruited by offering a free-of-charge screening for SARS-CoV-2 antibodies and is composed of both health care and non-health care workers of Ulm University hospital, Ulm, Germany. Initially, 2973 double- and triple-vaccinated study subjects were screened in this prospective cohort study between April 2021 and March 2022; 1461 study subjects had to be excluded due to a missing second or third vaccination dose at the time of blood sampling and/or incomplete/non-conclusive data records, ultimately resulting in the enrollment of 488 and 1024 individuals vaccinated twice (Vac2) or thrice (Vac3), respectively. Triple-vaccinated study subjects were further divided into two groups based on the time gap between the third vaccination and blood sampling (Vac3a: ≤12 weeks, n = 653 or Vac3b: >12 weeks, n = 371) to evaluate the short-term and long-term anti-S-RBD Ig antibody responses, respectively (Figure 1A) [20].

All study subjects were requested to fill in a basic questionnaire which included biometric information on age, sex, height and weight, smoking habits, previous infections with SARS-CoV-2, close contact with household members with SARS-CoV-2 infection, type and sequence of vaccines administered, date of vaccination, and vaccination-associated adverse events (AE). Written informed consent was obtained from all study subjects prior to entering the study.

### 2.2. Sample Acquisition and Assay

From all study subjects, 7.5 mL of venous blood was collected in serum tubes (S-Monovette^®^, Sarstedt, Nümbrecht, Germany) at each visit. A quantitative detection of Ig antibodies directed against the SARS-CoV-2 receptor-binding domain (RBD) of the spike protein subunit was performed using an electro-chemiluminescence immunoassay (Elecsys^®^ Anti-SARS-CoV-2 S, Roche, Basel, Switzerland) according to the manufacturer’s instructions. All anti-S-RBD Ig values were quantitatively expressed in units per milliliter (U/mL). The assay was considered positive if antibody concentrations exceeded 0.8 U/mL. This assay aids in detecting SARS-CoV-2 antibodies which result either from vaccination or natural infection. Therefore, an additional immunoassay for the qualitative detection of anti-nucleocapsid antigen antibodies was required to identify study subjects with a recent SARS-CoV-2 infection (Elecsys^®^ Anti-SARS-CoV-2, Roche). A cut-off index (COI) of ≥1.0 was interpreted as reactive (Figure 1B). In addition to study subjects testing positive for the presence of anti-nucleocapsid antigen antibodies, any study subject with a past positive SARS-CoV-2 PCR test result, optionally accompanied by symptoms of an upper respiratory infection, counted as a case of previous SARS-CoV-2 infection.

### 2.3. Data Organization and Statistical Analysis

Continuous variables are reported as median and interquartile range (IQR) while categorical variables are expressed as absolute numbers and their frequencies, respectively. If study subjects underwent anti-S-RBD Ig antibody quantification multiple times (>1 blood sample per subject), only the first assay result of the series was used for further analysis.

Prior to performing a regression analysis, the normal distribution of residuals was confirmed via quantile–quantile plot. First, a univariable linear regression analysis was applied to the datasets derived from the populations Vac2, Vac3a, and Vac3b to test the intragroup relationship between post-vaccination anti-S-RBD Ig antibody concentrations and the following independent variables: age, sex, body mass index (BMI), previous SARS-CoV-2 infection, household contacts with infected persons, type of vaccine combination, type of first vaccine, type of second vaccine, type of third vaccine (if applicable), occurrence of AE, and smoking. Only independent variables significantly associated with the dependent variable were accepted for subsequent multivariable linear regression analysis. A two-sided *p*-value of ≤0.05 was considered indicative of statistical significance. All statistical analyses were carried out using R, version 4.2.2.

## 3. Results

### 3.1. Description of Study Population

The double-vaccinated study population consisted of 488 study subjects (126 male, 362 female), of whom 1024 study subjects were already triple-vaccinated at the time of study enrollment (192 male, 832 female). All characteristics, anti-S-RBD Ig values, and results from the statistical analysis of the study subpopulations Vac2, Vac3a, and Vac3b are summarized in Table 1, Table 2, and Table 3, respectively.

**Figure 2:**
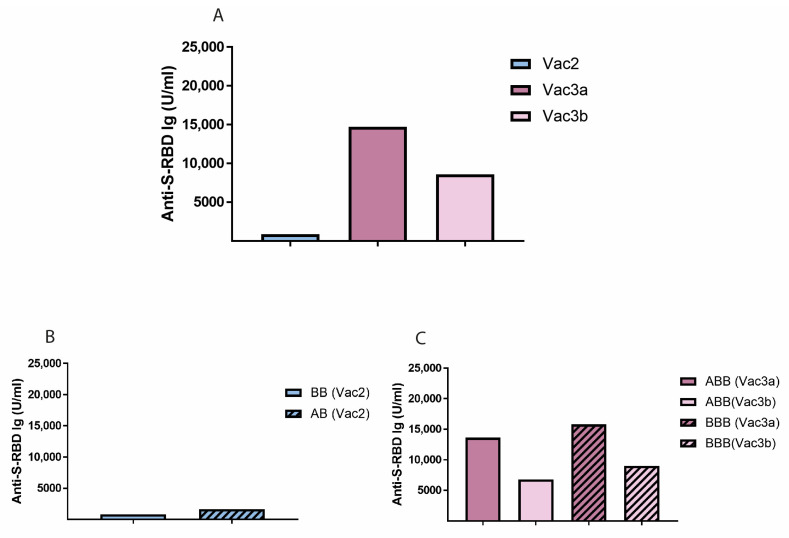
Total anti-S-RBD Ig concentrations in study groups Vac2, Vac3a, and Vac3b (**A**). Impact of vaccine combinations BB and AB in study group Vac2 (**B**) and BBB and ABB in study groups Vac3a and Vac3b (**C**) on anti-S-RBD Ig concentrations. Bar graphs represent median values of anti-S-RBD Ig concentrations. B, BNT162b2/BioNTech; A, ChAdOx1 n-CoV-19/AstraZeneca.

Among the double-vaccinated study subjects, a homologous strategy with two doses of BNT162b2 was the most frequently administered vaccination regimen (n = 407), followed by the hybrid regimen ChAdOx1 n-CoV-19/BNT162b2 (n = 62) (Figure 1C). The majority of triple-vaccinated study subjects had undergone a homologous regimen based on BNT162b2 (n = 472). Another 355 study subjects had received a heterologous regimen composed of a first vaccination with ChAdOx1 n-CoV-19 and two subsequent doses of BNT162b2, respectively (Figure 1D). Less frequent serial combinations of vaccines among double-vaccinated and triple-vaccinated study subjects are shown in Supplementary Data (Tables S1 and S2). The differences in anti-S-RBS Ig antibody concentrations between study groups Vac2, Vac3a, and Vac3b, which have been subdivided by the two most prevalent vaccination regimens in the respective group as stated above, are depicted in Figure 2.

### 3.2. Sex and BMI

The double-vaccinated study subjects developed similar anti-S-RBD Ig antibody concentrations irrespectively of their sex. Within 12 weeks following the third vaccination, the median anti-S-RBD Ig antibody concentration was 14,383 (8789.5–23,047.25) U/mL in female study subjects, while male study subjects achieved slightly higher median concentrations. Beyond 12 weeks, the anti-S-RBD Ig antibody concentrations declined in study subjects of both sexes, approximating median concentrations of 8546 U/mL (range: 4432.5–17,568.75 U/mL) in females and 9009 U/mL (range: 5085–14,394.5 U/mL) in males, respectively. Overall, no significant intragroup differences between female and male sex were detectable via univariable analysis.

In the double-vaccinated study subjects, anti-S-RBD Ig antibody concentrations were not found to be significantly influenced by their BMI. Within 12 weeks following their third vaccination, anti-S-RBD Ig antibody concentrations of overweight subjects, defined by a BMI ≥ 25, significantly exceeded anti-S-RBD Ig antibody concentrations of subjects below a BMI of 25. This significant difference persisted in the multivariable analysis A subgroup analysis revealed that, in particular, subjects with a BMI > 30 had anti-S-RBD Ig antibody concentrations that were significantly elevated compared with lean subjects, defined by a BMI < 18.5, who achieved the lowest values (*p* = 0.001).

### 3.3. Smoking

Among the double-vaccinated study subjects, there was no significant difference in the univariable analysis between smokers (n = 23) and non-smokers (n = 189). The remainder of the subjects had not disclosed information on their smoking status.

Altogether, 401 triple-vaccinated study subjects admitted to a current tobacco use, while the remainder either rated themselves as never-smokers or former smokers, respectively. Neither within nor beyond the 12 weeks following the third vaccination could a significant difference in anti-S-RBD Ig antibody concentrations be established.

### 3.4. Age Groups

Among the double-vaccinated study subjects, the median anti-S-RBD Ig antibody concentrations in the youngest cohort of <30 years reached a maximum of 1291 U/mL (range: 800–2012 U/mL), while the anti-S-RBD Ig values for all other age groups were consistently below 1000 U/mL. Testing the entire range of different age cohorts with univariable analysis yielded no significant differences.

With the exception of study subjects aged <30 years, who achieved significantly higher anti-S-RBD Ig antibody concentrations within the 12 weeks following the third vaccination compared with study subjects aged 30–39 (*p* = 0.046) and 40–49 (*p* = 0.033) years, respectively, the anti-S-RBD Ig antibody response was not affected by the study subjects’ age.

### 3.5. Vaccine Combinations and Single Agents

Dual immunization with BNT162b produced an antibody response significantly inferior to a heterologous regimen of ChAdOx1 n-CoV-19/BNT162b in the univariable analysis (*p* < 0.000). However, no statistical difference was seen in the multivariable analysis. Next, we separately examined the impact of the vaccines BNT162b and ChAdOx1 n-CoV-19 to dissect the contribution of each single vaccine to the anti-S-RBD Ig antibody response, depending on whether it had been used for the first or second immunization, respectively. Alternative vaccines such as mRNA-1273 and Ad26.COV2.S were excluded from the analysis since they were only administered in up to two study subjects per vaccine. Compared with BNT162b, ChAdOx1 n-CoV-19 elicited a significantly stronger anti-S-RBD Ig antibody response irrespectively of the timing within the vaccination schedule. However, this significant association was not observed in the subsequent multivariable analysis.

Within 12 weeks, the triple-vaccinated subjects with a BNT162b2-based homologous regimen exhibited significantly higher anti-S-RBD Ig antibody concentrations in the univariable analysis (*p* = 0.008). In the later course, the anti-S-RBD Ig antibody concentrations collectively dropped to lower concentrations which no longer differed.

In the multivariable analysis, the vaccination schedules containing BNT162b2 as the initial vaccine generated significantly higher anti-S-RBD Ig antibody concentrations compared with ChAdOx1 n-CoV-19, only if no more than 12 weeks since the third vaccination had elapsed (*p* = 0.002). On the other hand, BNT162b2 was outperformed by mRNA-1273 if administered as the third vaccine both in the univariable and multivariable analysis (both *p* < 0.000).

Beyond 12 weeks, the anti-S-RBD Ig antibody concentrations largely converged to similar concentrations. There was only a statistically significant difference between BNT162b2 and mRNA-1273 in the univariable (*p* < 0.000) and multivariable analysis (*p* = 0.001) if used as third vaccine.

### 3.6. Previous SARS-CoV-2 Infection

Serological screening combined with medical history pointed to a resolved infection with SARS-CoV-2 in 55 double-vaccinated study subjects. Compared with virus-naïve study subjects, those with a previous SARS-CoV2 exposure exhibited a nearly tenfold increase in their anti-S-RBD Ig antibody concentrations which, in both the univariable and multivariable analysis, achieved a robust statistical significance (both *p* < 0.000).

As before with the double-vaccinated study subjects, only a small number of the triple-vaccinated study subjects exhibited serological evidence of a previous SARS-CoV-2 infection or had reported a positive PCR test result. While in the univariable analysis a preceding infection with SARS-CoV-2 produced a significantly stronger anti-S-RBD Ig antibody response within the first 12 weeks after receiving the third vaccination (*p* = 0.003), this difference was no longer detectable in the multivariable analysis. Beyond 12 weeks, the anti-S-RBD Ig antibody concentrations remained elevated in study subjects who had convalesced from a SARS-CoV-2 infection, as opposed to a 3-fold reduction in non-infected study subjects, resulting in a significant difference between these two groups in the multivariable analysis (*p* < 0.000).

### 3.7. Household Contacts with a History of SARS-CoV-2 Infection

In total, 44 double-vaccinated study subjects had been sharing their living environment with a previously SARS-CoV-2-infected person, but tested negative for anti-nucleocapsid antigen antibodies themselves. In the univariable analysis, such exposed study subjects showed significantly increased anti-S-RBD Ig antibody readings compared with study subjects without a history of exposure (*p* < 0.000). This significant association could not be reproduced in subsequent multivariable analyses.

Of the 1.024 triple-vaccinated virus-naïve study subjects, 205 subjects had been in close contact with at least one household member with a previous SARS-CoV-2 infection. Again, the univariable analysis revealed that, within 12 weeks, exposed subjects developed significantly higher anti-S-RBD Ig antibody concentrations than non-exposed subjects (*p* = 0.027). This significant difference in anti-S-RBD Ig antibody response endured beyond the 12 weeks following the third vaccination (*p* = 0.002) but was not seen in the multivariable analysis.

### 3.8. Vaccine-Related AE

Among the double-vaccinated study subjects, the vast majority (n = 397) admitted to having experienced at least one vaccination-related AE of any grade of severity. In the univariable analysis, the anti-S-RBD Ig antibody concentrations did not significantly differ between study subjects with and without vaccination-related AE. Almost each triple-vaccinated study subject (n = 989) reported one or multiple complaints related to previous vaccinations, while only 35 subjects denied the occurrence of any AE. Based on the univariable analysis, the anti-S-RBD Ig antibody response was not significantly altered in the study subjects with AE, neither within the 12 weeks following their third vaccination nor beyond that period.

## 4. Discussion

Since the launch of clinical trials to examine promising candidate vaccines against SARS-CoV-2, many studies have been dedicated to characterizing the vaccine-induced humoral immune response in a variety of different clinical and epidemiological scenarios. Several studies have demonstrated that senescence is a major determinant of B and T cell immunoreactivity towards SARS-CoV-2 vaccines, leading to a dampened production of neutralizing antibodies in elderly people that can be restored by the application of a booster vaccination [21,22,23,24]. In general, standard vaccination schedules result in anti-S-RBD Ig concentrations being significantly lower in individuals older than 50–60 years as compared to younger age groups [25]. While this statement is true for BNT162b2, such age-related differences in post-boost antibody concentrations are not observed in individuals vaccinated with mRNA-1273 [26]. Indeed, our data show that the highest anti-S-RBD Ig concentrations were detected in study subjects younger than 30 years. Most of them had received BNT162b2 as their second and third vaccine dose, respectively.

As outlined in several publications, heterologous vaccine regimens combining an adenoviral vector-based and a mRNA-based vaccine are more effective at inducing a profound humoral immune response to SARS-CoV-2 [9,27,28,29]. This finding is endorsed by our results, showing a significantly higher increment of anti-S-RBD Ig concentrations in double-vaccinated subjects with a heterologous vaccine regimen. Interestingly, the opposite effect was observed in triple-vaccinated study subjects with a heterologous vaccination, including ChAdOx1 n-CoV-19 and two doses of BNT162b2a, in whom anti-S-RBD-Ig antibody concentrations were significantly lower compared to those with a homologous BNT162b2-based schedule. By contrast, Gareayaghi et al. documented similar levels of neutralizing anti-S-RBD Ig antibodies in individuals with two doses of CoronaVac or BNT162b2, respectively, all of whom had opted for a booster vaccination with BNT162b2 [30]. In addition, we could show that, if delivered as the final vaccine in triple-vaccinated study subjects, mRNA-1273 was capable of stimulating a significantly stronger anti-S-RBD-Ig antibody response compared with BNT162b2 and ChAdOx1 n-CoV-19, remaining stable even beyond 12 weeks from vaccination. This finding is supported by a study which encountered the most durable anti-S-RBD-Ig immune response in individuals who had received mRNA-1273 as their primary vaccination [31].

It is well documented that following the recovery from a SARS-CoV-2 infection subsequent vaccination can significantly reinforce the antibody response, providing a robust and sustained level of humoral immunity [32,33]. This finding was ascertained by our study results, which could reproduce significantly increased anti-S-RBD Ig antibody concentrations in both double- and triple-vaccinated study subjects with a history of SARS-CoV-2 infection. Notably, in the triple-vaccinated subjects, their antibody concentrations continued to rise beyond the 12 weeks following vaccination, supporting the observation of a long-lived immunostimulatory effect mediated by a natural SARS-CoV-2 infection. Notably, even study subjects without serological evidence of a past infection exhibited an exaggerated post-vaccination response if they had been sharing their living environment with a person with a history of SARS-CoV-2. It remains a matter of speculation whether in some of those study subjects a silent transmission of SARS-CoV-2 had gone unnoticed by anti-nucleocapsid antigen antibody screening, which only upon vaccination induced an appreciable increase in anti-S-RBD Ig concentrations. In the literature, the detection of anti-nucleocapsid antigen antibodies following infection has been described to last for a median time of 8 months before “sero-reversion” (loss of nucleocapsid seropositivity) occurs [34]. In contradiction to previously published studies [35,36], the odds of experiencing vaccination-related AE were not correlated with the levels of anti-S-RBD-Ig antibodies in neither the double- nor the triple-vaccinated study subjects.

As secondary factors modulating the vaccine-induced antibody response to SARS-CoV-2, we gathered additional information on the BMI and smoking habits of the study subjects. Contrary to the literature [10,37], the active smokers did not seem to build up significantly lower anti-S-RBD Ig antibody concentrations compared with the study subjects who had quit smoking or were never-smokers, which could be explained by the overall small number of active smokers in each group. Strikingly, in our study, overweight triple-vaccinated subjects with a BMI > 30 demonstrated significantly higher anti-S-RBD Ig antibody concentrations compared with normal-weight study subjects, which conflicts with several other available studies [12,38]. However, the data from an Italian longitudinal study on the efficacy of BNT162b286 in a cohort of 86 health care workers indicate that a reduction in antibody levels was primarily associated with central obesity rather than with BMI [39].

The particular strength of our study relates to a relatively large number of study subjects, resulting in a meaningful statistical power. However, our study also comes with limitations: firstly, the study population cannot be considered a representative cross-section of the average population due to the preponderance of female subjects typically encountered in health care-related professions, and age groups covering the extremes were either underrepresented or missing. Furthermore, the percentage of subjects with a previous SARS-CoV-2 infection could have been skewed, owing to a higher likelihood of contagious contacts in the healthcare setting. Secondly, using the anti-S-RBD Ig antibody concentrations as the only immunological read-out merely approximates the protective immunity.

## 5. Conclusions

Taken together, this study provides information on how the humoral immunogenicity of the vaccines BNT162b2, mRNA-1273, and ChAdOx1 n-CoV-19 can be modulated by combining different vaccines together, or with the succession of the single vaccine agents in a population of predominantly healthy individuals at different stages of active immunization. In addition, it corroborates the results of former studies, pointing to the superiority of a hybrid humoral immunity following SARS-CoV-2 infection. This may be of value for future cohort selection and vaccination strategies.

## Figures and Tables

**Table 1:** Basic characteristics, anti-S-RBD Ig antibody values, and results from univariable/multivariable regression analysis in study population Vac2. Levels of significance: ** *p* ≤ 0.01; *** *p* ≤ 0.001.

	All (n = 488)		Linear Regression Analysis, Univariable			Linear Regression Analysis, Multivariable		
Variable	Data Available, n	Anti-S-RBD Ig Antibody Value (U/mL) (Median, IQR)	Estimate	T Value	*p*-Value	Estimate	T Value	*p*-Value
Age (year-old)								
- <30	83	1291 (800–2012)						
- 30–39	115	941 (594–1435)	−1281	−0.001	0.999			
- 40–49	109	706 (471–1369)	−1072.374	−1.235	0.217			
- 50–59	117	881 (382.75–1603.5)	466.211	0.546	0.585			
- 60	64	736 (247.5–1299.5)	−519.517	−0.526	0.599			
Sex								
- Male	126	866 (497.5–1498.5)						
- Female	362	932.5 (500.25–1.583.75)	160.7	0.262	0.794			
BMI								
- <18.5	12	741 (655–1016)						
- 18.5–24.9	296	938.5 (533.5–1631.5)	1650	0.946	0.345			
- 25–29.9	123	890 (482.75–1442)	1774	0.990	0.323			
- ≥30	56	669 (425.75–1344)	3069	1.629	0.104			
Smoking								
- Never- or ex-smoker	189	1086 (592.5–2849.5)						
- Current smoker	23	1022 (451–2248)	1078.0	0.621	0.535			
Previous SARS-CoV-2 infection								
- Yes	55	15,230 (4853–25,000)						
- No	433	795 (481–1295)	13,360.5	22.122	<0.000 (***)	17,317.7	19.759	<0.000 (***)
Contact with infected household members								
- Yes	44	1.805 (720–22,289.5)						
- No	113	987.5 (486.25–2085.25)	5243.8	3.596	<0.000 (***)	−969.3	−1.167	0.245
Vaccine combination								
- BB	407	816 (481–1.394)						
- AB	62	1687 (850–4971.5)	4221.7	5.674	<0.000 (***)			
First vaccine								
- B	407	816 (481–1394)						
- A	78	1435 (754–6888)	4318.6	6.192	<0.000 (***)	855.4	1.130	0.2602
Second vaccine								
- B	470	888 (499.25–1555)						
- A	16	946 (428.25–14,071.5)	4121.3	2.788	0.006 (**)	195.2	0.150	0.8807
Vaccine-related adverse events								
- Yes	397	951 (544.5–1571.5)						
- No	91	706 (388.5–1391)	−81.5	−0.118	0.906			

IQR, interquartile range; A, ChAdOx1 n-CoV-19/AstraZeneca; B, BNT162b2/BioNTech

**Table 2:** Basic characteristics, anti-S-RBD Ig antibody values, and results from univariable/multivariable regression analysis in study population Vac3a. Levels of significance: * *p* ≤ 0.05; ** *p* ≤ 0.01; *** *p* ≤ 0.001.

	All (n = 653)		Linear Regression Analysis, Univariable			Linear Regression Analysis, Multivariable		
Variable	Data Available, n	Anti-S-RBD Ig Antibody Value (U/mL) (Median, IQR)	Estimate	T Value	*p*-Value	Estimate	T Value	*p*-Value
Age (year-old)								
- <30	129	16,189 (10,184.5–25,000)						
- 30–39	128	13,597 (8880–21,164.5)	−1848.2	−2.000	0.046 (*)	−1852.9	−2.050	0.041 (*)
- 40–49	148	13,795,5 (7869.25–21,996.25)	−1910.0	−2.140	0.033 (*)	−2860.3	−3.199	0.001 (**)
- 50–59	172	16,060 (9526.5–23,179.5)	−468.9	−0.543	0.587	−1111.5	−1.268	0.205
- >60	76	12,889 (9439.5–20,558.5)	−1713.1	−1.600	0.110	−2042.1	−1.925	0.055
Sex								
- Male	116	16,675 (11,293.5–24,408.5)						
- Female	537	14,383 (8789.5–23,407.5)	−1088.4	−1.435	0.152			
BMI								
- <18.5	13	9937 (5252–15,135)						
- 18.5–24.9	364	14,121 (8465.5–21,915.5)	4416	2.143	0.033 (*)	4610.5	2.277	0.023 (*)
- 25–29.9	175	14,984.5 (9955.75–22,838.75)	5182	2.468	0.014 (*)	5224.3	2.523	0.012 (*)
- ≥30	98	18,813 (11,715–25,000)	7250	3.364	0.001 (***)	7598.2	3.561	<0.000 (***)
Smoking								
- Never- or ex-smoker	579	14,739 (9315–23,596)						
- Current smoker	74	14,403.5 (7837.5–19,939.25)	−832.9	−0.91	0.363			
Previous SARS-CoV−2 infection								
- Yes	50	18,937 (12,770.75–25,000)						
- No	603	14,427 (8800–22,600)	3288.4	3.033	0.003 (**)	2173.7	1.950	0.052
Contact with infected household members								
- Yes	119	16,868 (9667–25,000)						
- No	534	14,383 (9010–22,234.5)	1658.4	2.213	0.027 (*)	1076.6	1.389	0.165
Vaccine combination								
- ABB	271	13,641 (7985.75–19,337.75)						
- BBB	226	15,793 (9677–25,000)	−1754.2	−2.672	0.008 (**)			
First vaccine								
- B	251	16,187 (9879.75–25,000)						
- A	391	14,007 (8758–20,626.5)	−1665.7	−2.792	0.005 (**)	−1775.5	−3.048	0.002 (**)
- M	11	20,813 (11,825–25,000)	1588.0	0.699	0.485	−626.4	−0.283	0.778
Second vaccine								
- B	557	14,638 (9253.5–23,180.75)						
- A	75	14,565.5 (8758–21,181.25)	−691.8	−0.758	0.449			
- M	21	17,056 (9480–25,000)	1041.4	0.631	0.528			
Third vaccine								
- B	569	14,310 (8758–21,728)						
- A	2	10,489.5 (8463.25–12,515.75)	−4152.0	−0.803	0.423	−2461.7	−0.487	0.626
- M	82	20,797.5 (11,477.5–25,000)	3950.1	4.578	<0.000 (***)	3921.2	4.524	<0.000 (***)
Vaccine-related adverse events								
- Yes	620	14,800 (9315–23,182)	1135	0.857	0.392			
- No	33	12,275,5 (6372.5–21,725.75)						

IQR, interquartile range; A, ChAdOx1 n-CoV-19/AstraZeneca; B, BNT162b2/BioNTech; M, mRNA-1273/Moderna.

**Table 3:** Basic characteristics, anti-S-RBD Ig antibody values, and results from univariable/multivariable regression analysis in study population Vac3b. Levels of significance: ** *p* ≤ 0.01; *** *p* ≤ 0.001.

	All (n = 371)		Linear Regression Analysis, Univariable			Linear Regression Analysis, Multivariable		
Variable	Data Available, n	Anti-S-RBD Ig Antibody Value (U/mL) (Median, IQR)	Estimate	T Value	*p*-Value	Estimate	T Value	*p*-Value
Age (year-old)								
- <30	67	8645 (6210–15,693)						
- 30–39	88	7986 (4291.25–18,578.75)	16.27	0.013	0.990			
- 40–49	73	9138 (5139–19,087)	681.07	0.506	0.613			
- 50–59	103	9465 (4164–17,568.75)	285.93	0.229	0.819			
- >60	40	8300 (4384–15,453)	−798.58	−0.502	0.616			
Sex								
- Male	76	9009 (5805–14,394.5)						
- Female	295	8546.5 (4432.5–17,568.75)	25.26	0.025	0.980			
BMI								
- <18.5	6	10,609.5 (5559.25–20,610.5)						
- 18.5–24.9	210	7772.5 (3782–16,267.25)	−2797	−0.853	0.394			
- 25–29.9	99	9126.5 (5346.5–18,072.75)	−1453	−0.436	0.663			
- ≥30	55	10,224 (6834–17,765)	−636	−0.187	0.852			
Smoking								
- Never- or ex-smoker	327	8595 (4825–17,608.5)						
- Current smoker	44	8214 (3949–13,985)	−1452	−1.141	0.254			
Previous SARS-CoV-2 infection								
- Yes	55	23,307 (12,215–25,000)						
- No	316	7644 (4145–14,323)	8726.3	8.181	<0.000 (***)	6347.2	4.066	<0.000 (***)
Contact with infected household members								
- Yes	86	12,313.5 (5749.25–23,699.25)						
- No	285	7917.5 (4341.5–15,827.75)	2977.1	3.089	0.002 (**)	−741.5	−0.692	0.490
Vaccine combination								
- ABB	84	6798.5 (4056.25–14,611.5)						
- BBB	246	9011 (4525.5–16,621.5)	−1175.8	−1.182	0.238			
First vaccine								
- B	257	9126.5 (4692.75–18,358.75)						
- A	110	7297 (4127.75–15,608.25)	−1010.7	−1.119	0.264			
- M	4	8439 (6891.75–10,111.75)	−2865.4	−0.717	0.474			
Second vaccine								
- B	346	8631 (4487.5–17,608.5)						
- A	18	7837 (3980.5–14,496.75)	−674.9	−0.352	0.725			
- M	7	7174 (6844.5–10,284.5)	−2460.4	−0.812	0.417			
Third vaccine								
- B	348	8295 (4404–16,226)						
- A	3	4333 (2167–5947)	−6825.9	−1.515	0.131	−5761.4	3.296	0.167
- M	20	18,608.5 (11,554–25,000)	6745.2	3.775	<0.000 (***)	5492.3	−1.384	0.001 (**)
Vaccine-related adverse events								
- Yes	369	8589.5 (4485.75–16,663.25)						
- No	2	15,448.5 (10,771.75–20,125.25)	−4373	−0.778	0.437			

IQR, interquartile range; A, ChAdOx1 n-CoV-19/AstraZeneca; B, BNT162b2/BioNTech; M, mRNA-1273/Moderna.

## Data Availability

Data is contained within the article or Supplementary Material.

## Supplementary Materials

The following supporting information can be downloaded at https://www.mdpi.com/article/10.3390/vaccines11091467/s1, Table S1: Continued listing of vaccine combinations in double vaccinated study subjects; Table S2: Continued listing of vaccine combinations in triple vaccinated study subjects.
