# Supplementary material for: A Systematic Evaluation of the SARS-CoV-2 Vaccine-Induced Anti-S-RBD-Ig Response in a Population of Health Care Workers
Source: Vaccines (Basel). 2023 Sep 7;11(9):1467. doi: 10.3390/vaccines11091467 (PMC10537165; doi:10.3390/vaccines11091467)
Supplement: Supplementary file 1 [file vaccines-11-01467-s001.zip › vaccines-2585140-supplementary.pdf]

Table S1. Continued listing of vaccine combinations in double vaccinated study subjects;.

**No. of double vaccinated study subjects** (n = 488, % of total)	**1st vaccination**	**2nd vaccination**
16 (3.28)	ChAdOx1 n-CoV-19	ChAdOx1 n-CoV-19
2 (0.41)	mRNA-1273	mRNA-1273
1 (0.21)	Ad26.COV2.S	BNT162b2

Table S2: Continued listing of vaccine combinations in triple vaccinated study subjects.

**No. of triple vaccinated study subjects** (n = 1.024, % of total)	**1st vaccination**	**2nd vaccination**	**3rd vaccination**
73 (7.13)	ChAdOx1 n-CoV-19	ChAdOx1 n-CoV-19	BNT162b2
39 (3.81)	ChAdOx1 n-CoV-19	BNT162b2	mRNA-1273
33 (3.22)	BNT162b2	BNT162b2	mRNA-1273
19 (1.86)	ChAdOx1 n-CoV-19	ChAdOx1 n-CoV-19	mRNA-1273
9 (0.88)	mRNA-1273	mRNA-1273	BNT162b2
8 (0.78)	ChAdOx1 n-CoV-19	mRNA-1273	BNT162b2
6 (0.59)	mRNA-1273	mRNA-1273	mRNA-1273
5 (0.49)	ChAdOx1 n-CoV-19	mRNA-1273	mRNA-1273
3 (0.29)	BNT162b2	BNT162b2	ChAdOx1 n-CoV-19
1 (0.10)	ChAdOx1 n-CoV-19	BNT162b2	ChAdOx1 n-CoV-19
1 (0.10)	ChAdOx1 n-CoV-19	ChAdOx1 n-CoV-19	ChAdOx1 n-CoV-19
